# Investigations of the α-Olefin Polymerization Process Using the Classic α-Diimine Nickel Catalyst

**DOI:** 10.3390/polym18080961

**Published:** 2026-04-15

**Authors:** Ying Wang, Jingjing Lai, Zhihui Song, Rong Gao, Qingqiang Gou, Bingyi Li, Gang Zheng, Randi Zhang, Qiang Yue, Yuanning Gu

**Affiliations:** Department of Polyethylene, SINOPEC (Beijing) Research Institute of Chemical Industry Co., Ltd., Beijing 100013, China; laijj.bjhy@sinopec.com (J.L.); songzhh.bjhy@sinopec.com (Z.S.); gaor.bjhy@sinopec.com (R.G.); gouqq.bjhy@sinopec.com (Q.G.); liby.bjhy@sinopec.com (B.L.); zhenggang.bjhy@sinopec.com (G.Z.); zhangrd.bjhy@sinopec.com (R.Z.); yueq.bjhy@sinopec.com (Q.Y.); guyn0423.bjhy@sinopec.com (Y.G.)

**Keywords:** α-olefin polymerization, α-diimine Ni catalyst, quasi-living polymerization, chain-walking, chain structure regulation

## Abstract

This work provides a comprehensive exploration of α-olefin polymerization characteristics catalyzed by the classic α-diimine Ni catalyst. The polymerization process exhibited quasi-living behaviour, and a reaction kinetic model for the monomer coordination–insertion process was established. It was observed that the reaction exhibits living polymerization features during the first 10 min, and the coordination–insertion rate constant was determined to be 1.08 L·mol^−1^·s^−1^ at 30 °C. The regulation rules for factors including co-catalyst amount, monomer concentration, polymerization temperature, monomer type on the molecular weight, molecular weight distribution and chain structure of poly(α-olefin)s were clarified. The co-catalyst (methylaluminoxane) primarily served to activate the catalyst without inducing a chain transfer effect, suggesting that chain stagnation is likely the primary cause of the deviation from typical living polymerization behaviour. Based on temperature-controlled experiments, the activation energy for the coordination–insertion reaction was calculated to be 28.40 kJ·mol^−1^ through GPC curve analysis. The kinetic model established in this study, along with the revealed chain branching rules, provides a theoretical foundation for the design of poly(α-olefin)s with novel structures and functions.

## 1. Introduction

**Figure 1:**
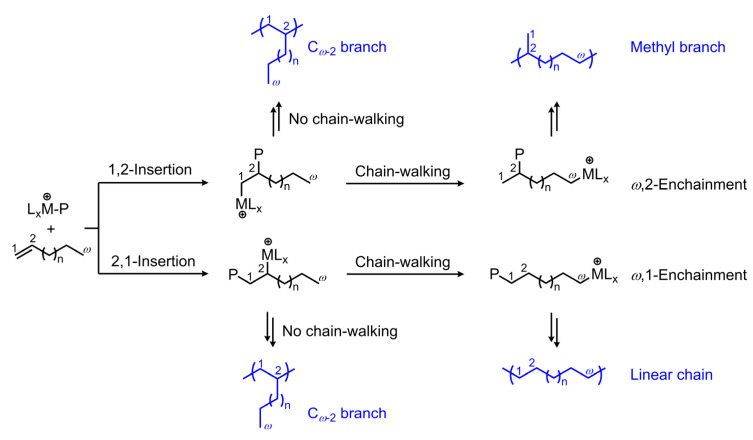
Chain-walking mechanism of α-olefin polymerization by α-diimine-type catalysts.

As the general polyolefin market gradually becomes saturated, there is an urgent need for the development of high-value-added polyolefin products with specific chain structures [1,2,3,4]. The chain-walking characteristics of late transition metal catalysts provide an ingenious way to customize polyolefins with desirable chain structures and properties [5,6,7,8,9]. Unlike traditional poly(α-olefin)s with uniform branched structures (C*_ω_*_-2_ side chains, where *ω* indicates the number of carbon atoms in α-olefins), poly(α-olefin) products prepared using α-diimine-type late transition metal catalysts typically display a wider variety of branched chain structures (C*_ω_*_-2_ chains, methyl groups, etc.). A classic α-diimine Ni catalyst and a representative metallocene Zr catalyst were presented to provide a direct comparison of their respective characteristics during 1-octene polymerization (Table 1) [10,11]. Due to their regular branching and relatively lower molecular weights, poly(α-olefin)s produced by metallocene catalysts are typically employed as lubricant base oils. In contrast, the proportion of the three typical chain compositions produced by α-diimine nickel catalysts can be adjusted, enabling the preparation of polymers ranging from amorphous to semicrystalline forms [12,13,14]. Figure 1 illustrates the typical chain-walking polymerization process of α-olefins based on α-diimine-type catalysts. In the case of 1,2-insertion, methyl branches can be formed if the active metal centre predominantly walks towards the *ω*-carbon after insertion; 2,1-insertion promotes *ω*,1-chain growth, resulting in linear semicrystalline PE-like segments [13,15]; if there is no chain-walking following monomer insertion, the regular C*_ω_*_-2_ branches will appear in the poly(α-olefin)s. The type and content of different branches play a crucial role in influencing the physical and mechanical properties of the poly(α-olefin) products, thereby determining their application performance. The C*_ω_*_-2_ branch is more likely to disrupt the crystallinity of the main chain than the methyl branch. This is because the methyl branch can be assembled into the polyethylene crystal lattice, whereas the C*_ω_*_-2_ branch cannot [16]. Therefore, poly(α-olefin)s based on α-diimine-type catalysts usually appear as amorphous or semicrystalline products, highlighting their potential utility as thermoplastic elastomers [11,17,18].

**Figure 2:**
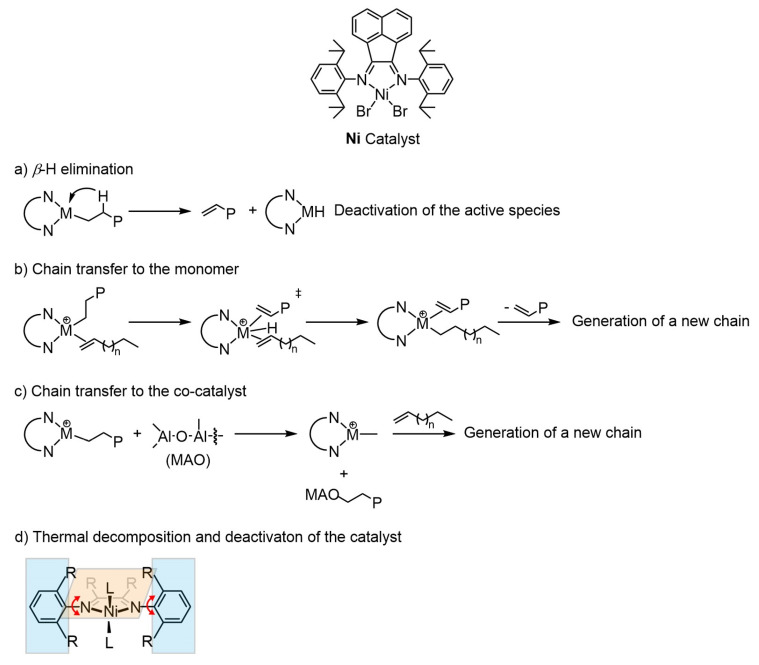
General routines for the chain termination during the α-olefin coordination polymerization process.

During the α-olefin coordination polymerization process using α-diimine-type catalysts, *β*-H elimination, chain transfer and the thermal effect may cause the termination of the original chains, the generation of new chains or the deactivation of the active centre. In detail, the formation of Ni-H bonds during the *β*-H transfer process from the polymer chain to the active centre will directly lead to the deactivation of the catalyst (Figure 2a) [19]. For the chain transfer process to the monomer or the co-catalyst, the original polymer chain can be terminated, and the generation of new chains can be triggered (Figure 2b,c) [9]. In addition, α-diimine-type catalysts can also be deactivated by environmental factors (such as elevated temperature or water oxygen). The C-N bond of the ligand structure may rotate at elevated temperatures (above 60 °C), thus causing the activation of the adjacent C-H bond along with the aggravation of the chain transfer process (Figure 2d). Ultimately, the in situ generation of Ni-H species may occur [20].

This work deeply investigated the α-olefin polymerization characteristics using a classic α-diimine Ni catalyst (Figure 2). The polymerization conditions will affect the chain-initiation, chain-growth, chain-walking, chain-transfer, chain-termination, catalyst stability and other processes in the polymerization, thereby determining the properties of the polymer product. The polymerization of α-olefins mediated by α-diimine catalysts has received considerable attention in existing studies [11,12,13,14,15,17,21,22,23,24]. However, research on the kinetics for this reaction remains relatively unexplored. Herein, a classic α-diimine nickel catalyst was used for the present study. The characteristics of α-olefin polymerization catalyzed using this catalyst were systematically discussed in relation to the polymerization behaviours documented in the literature. Herein, we first studied the polymerization kinetics to provide a basis for the regulation of the polymer chain structure. Furthermore, various polymerization parameters, including co-catalyst amount, monomer concentration, polymerization temperature and monomer type, were studied to clarify the regulation rules for polymer properties. The chain structure, molecular weight and molecular weight distribution under different conditions were summarized and analyzed. Our work will provide a research foundation for the development of functional polyolefins using the α-diimine-type catalysts.

## 2. Materials and Methods

α-Olefins were purchased from J&K Scientific Ltd., Beijing, China and dried over molecular sieves. Toluene was purchased from Hangzhou Chemical Reagents Company, Hangzhou, China and purified by the MB SPS-800X (MBRAUN, Garching, Germany) solvent purification system before use. Methylaluminoxane (10 wt% in toluene solution) (MAO) was purchased from Albemarle Corporation, Charlotte, NC, USA and used directly. Ethylaluminum sesquichloride (0.4 M, hexane) (Al_2_Et_3_Cl_3_) was purchased from Sigma-Aldrich, St. Louis, MO, USA and used directly. The α-diimine Ni catalyst was synthesized in the laboratory using procedures based on previously published methods [25]. All reagents were placed in a N_2_ box.

The ^1^H NMR spectrum was recorded on a Bruker 400 MHz spectrometer at room temperature, with tetramethylsilane (TMS) as the internal standard (Bruker Co., Billerica, MA, USA). Molecular weight, molecular weight distribution and branching density of polymer products were tested by a gel permeation chromatograph equipped with an IR5 infrared detector in 1,2,4-trichlorobenzene solution at 150 °C (Polymer Char, Inc., Valencia, Spain). Four chromatographic columns with an outer diameter of 3/8″ and a length of 33 cm were used.

The polymerization reactions of α-olefins were carried out in a Schlenk flask (Synthware, Chongqing, China) equipped with magnetic stirring. The flask needed to be baked in a constant temperature drying box at 100 °C for 6 h, then purged with argon three times. The flask was kept at the required polymerization temperature, and then the monomer, co-catalyst solution and solvent preheated to the reaction temperature were added to the flask. Afterwards, the α-diimine Ni catalyst solution, preheated to the reaction temperature, was injected to trigger the polymerization. After reaching the required reaction time, the polymerization was immediately quenched by a HCl/EtOH (1/9, V/V) solution. Then, the poly(α-olefin)s were washed and precipitated with ethanol and then baked for 8 h to 12 h in a 60 °C vacuum oven to achieve a constant weight. Monomer conversion for kinetic analysis was calculated based on the weight of the isolated polymer.

## 3. Results and Discussion

### 3.1. Polymerization Kinetics of 1-Hexene

Some α-diimine-type catalysts exhibit living polymerization characteristics when used in the α-olefin polymerization. This feature allows for the preparation of polymers with controllable molecular weights and narrow dispersities. Herein, the classic α-diimine Ni catalyst was adopted to investigate the polymerization kinetics for 1-hexene. The results indicated that the Ni catalyst displayed quasi-living characteristics during the polymerization process. A typical living polymerization is characterized by several key features: the chain growth rate follows first-order kinetics with respect to both the concentration of monomer and the active centre ($\frac{dc}{dt}\;=\;-{\mathrm{kc}}_{\mathrm{cata.}}c$, $\ln\frac{c_{0}}{c_{t}}\;=\;{\mathrm{kc}}_{\mathrm{cata.}}t$); i.e., ln(*c*_0_/*c_t_*) is linearly related to the polymerization time *t*; the polymerization degree can be predicted by the monomer conversion; the molecular weight distribution remains narrow (~1.0) and conforms to a Poisson distribution; the lifetime of the polymer chain is long enough to remain active even after all the monomer has been completely consumed.

**Figure 3:**
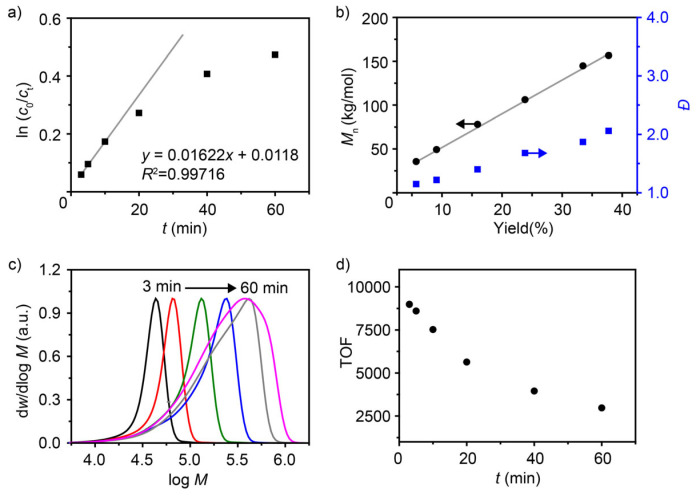
Kinetic plots for the polymerization process. (**a**) Semilogarithmic plots of monomer conversion versus polymerization time. (**b**) Dependence of the number-average molecular weight and dispersity of polymers on the monomer conversion. (**c**) GPC curves of poly(1-hexene)s for the kinetic studies. (**d**) The TOF (calculated after reaction termination) versus polymerization time. Polymerization conditions: polymerization times are 3, 5, 10, 20, 40, 60 min, 30 °C, [1-hexene] = 2 M, Ni catalyst 10 μmol, MAO (Al/Ni = 230), toluene, total volume = 40 mL.

In this 1-hexene polymerization system, the plot of ln(*c*_0_/*c_t_*) versus polymerization time *t* exhibits a linear relationship during the initial 10 min. However, the slope gradually decreases, and the plots deviate from linearity after 20 min (Figure 3a). The initial linear relationship suggests that the activation process of the catalyst occurs relatively quickly. The apparent first-order kinetic constant *k* was estimated to be 1.08 L·mol^−1^·s^−1^ (*R*^2^ = 0.9972), according to the linear relationship during the first 10 min. The number-average molecular weight of the polymer and the monomer conversion are essentially linear (Figure 3b). The GPC curves indicate that the molecular weight distribution broadens from 1.15 to 2.06 as polymerization time increases (Figure 3c). Specifically, the portion of the lower-molecular-weight part on the left side of the peak gradually increases. In contrast, the shape of the right side of the peaks (higher-molecular-weight part) remains basically unchanged, consistent with the random distribution characteristic of a living polymerization process. The monomer turnover frequency (TOF, the number of monomers consumed per unit time on a unit active site) gradually decreases over 60 min (Figure 3d). Due to the decrease in monomer concentration over time, the lifetime of the catalyst cannot be evaluated based on the TOF. These polymerization characteristics indicate that this polymerization was conducted in a quasi-living manner. The derivation from typical living polymerization may be caused by the cumulation of *β*-H elimination, chain transfer, and/or chain stagnation with an increase in the system viscosity [21]. A typical living polymerization may occur at very low temperature (−10 °C) and low concentration [22,26].

### 3.2. Effect of Co-Catalyst (MAO) Dosage on 1-Hexene Polymerization

**Figure 4:**
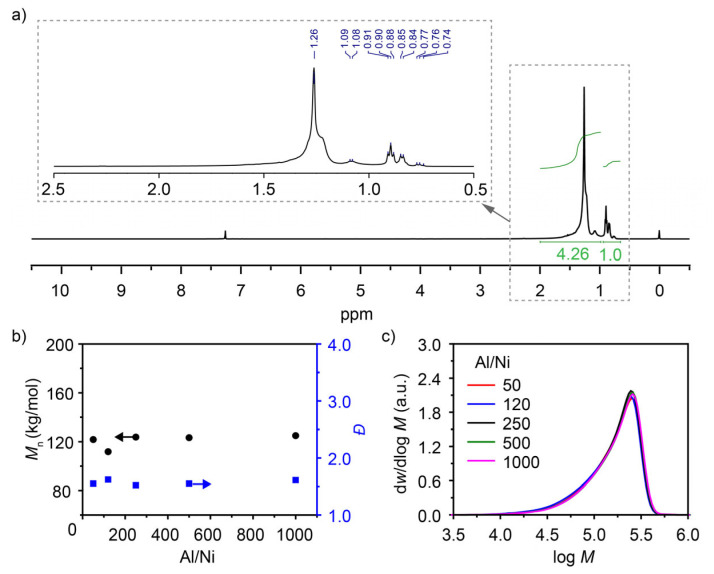
(**a**) ^1^H NMR spectrum of the sample reacting for 60 min in the polymerization kinetic study. (**b**) Dependence of the number-average molecular weight and dispersity of polymers on the amount of co-catalyst. (**c**) GPC curves of poly(1-hexene)s prepared with different amounts of the co-catalyst (MAO). Polymerization conditions of (**b**,**c**): 30 °C, 10 min, [1-hexene] = 4 M, Ni catalyst 10 μmol, MAO (Al/Ni = 50, 120, 250, 500, 1000), toluene, total volume = 40 mL.

To further clarify the reason why the polymerization system deviated from a typical living polymerization, the ^1^H NMR analysis of the polymer product and the effect of co-catalyst dosage were investigated. It is well known that double bonds can form during the *β*-H elimination or the chain transfer to monomer process. The sample after 60 min of reaction was subjected to characterization. No signal corresponding to -C=CH_2_ was observed in the hydrogen shift range of 4~6 ppm (Figure 4a). Therefore, it is speculated that the *β*-H elimination and chain transfer to monomer can be left out in this polymerization system.

The study of the MAO dosage effect helps determine whether the chain transfer to the co-catalyst process occurs. In the Ni catalyst/MAO system, MAO dosages with Al/Ni ratios of 50, 120, 250, 500, and 1000 were investigated. The polymerization activity (yield of approximately 1.64 g per sample) did not vary with the Al/Ni ratio. Moreover, the molecular weight (*M*_n_~121,000 g mol^−1^) and molecular weight dispersity (*Ɖ*~1.57) remained largely unchanged with varying Al/Ni ratios (Figure 4b). A single peak with a generally consistent shape was observed in the GPC spectra (Figure 4c). These results indicate that the co-catalyst only exhibits the active function for the catalyst, and an Al/Ni ratio of 50 equivalents is sufficient for the entire activation process. The chain transfer process to the co-catalyst can be considered negligible in this system [21,23,27]. Consequently, the deviation from typical living polymerization in this system is likely due to the chain stagnation. Potential causes of the chain stagnation include: (1) decomposition and deactivation of the catalyst during polymerization; (2) an increase in the monomer coordination–insertion resistance as the polymer chain lengthens during polymerization, which may make the coordination–insertion difficult or even stop, or it may cause the active centres to undergo chain-walking rather than chain growth. These factors may be further clarified in future work using techniques such as X-ray absorption spectroscopy [28,29].

### 3.3. Effect of Monomer Concentration on 1-Hexene Polymerization

**Figure 5:**
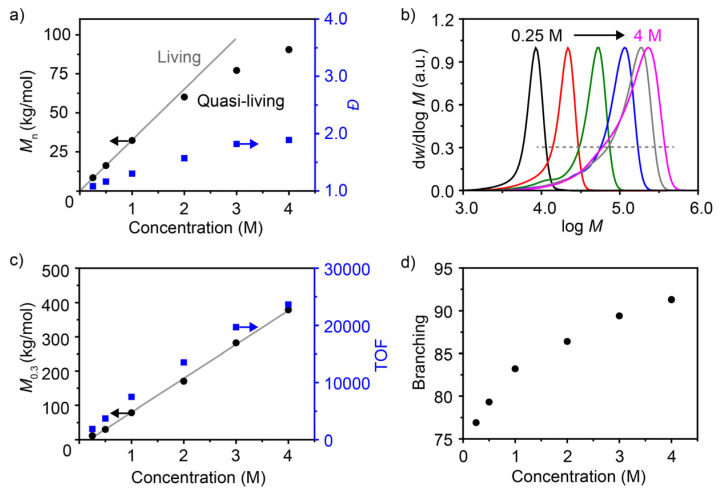
Polymerization of 1-hexene with different monomer concentrations. (**a**) Dependence of the number-average molecular weight and dispersity of polymers on the monomer concentration. (**b**) GPC curves of poly(1-hexene)s. (**c**) The molecular weight *M*_0.3_ at d*w*/dlog *M* = 0.3 and the TOF (calculated after reaction termination) versus monomer concentration. (**d**) The change in polymer branching with monomer concentration. Polymerization conditions: monomer concentrations are 0.25, 0.5, 1, 2, 3, and 4 M, 10 min, 30 °C, Ni catalyst 10 μmol, Al_2_Et_3_Cl_3_ (Al/Ni = 200), toluene, total volume = 40 mL.

The concentration of α-olefins has a significant impact on the polymerization process and the resultant polymer [11]. Coates et al. found that when the concentration of 1-decene was decreased from 3.53 M to 0.1 M in the α-diimine Ni catalytic system, the carbon atom content in the main chain of poly (1-decene) increased from 52% to 77%, accompanied by an increase in the melting point from 63 °C to 106 °C [13]. In our system, 1-hexene was adopted as the polymeric monomer, and the Ni catalyst/Al_2_Et_3_Cl_3_ was used as the catalytic system for the investigation. As the monomer concentration increased from 0.25 M to 4 M, the number-average molecular weight rose from 8467 g mol^−1^ to 90,500 g mol^−1^. In the concentration range of 0.25 M to 1 M, the polymerization of 1-hexene follows a typical living polymerization behaviour. However, as the concentration continued to increase to 4 M, the molecular weight gradually deviated from linear growth, along with an increase in the dispersity (Figure 5a). Upon examination of the GPC curves, it is apparent that the lower-molecular-weight part gradually increased as the monomer concentration rose, with a bulge peak appearing (Figure 5b). These observations may be rationalized by the chain stagnation process [18,25]. Random values (d*w*/dlog *M* = 0.3) were taken from the right side of the GPC curve (conforms to the random distribution of molecular weight during the polymerization process) and then plotted against concentration. It was found that the molecular weight *M*_0.3_ is linearly correlated with the concentration, indicating that the growth process of an individual polymer chain possesses living features. The TOF showed a progressive increase with rising concentration (Figure 5c). Furthermore, the branching degree of the polymer increased with increasing monomer concentration (from 77/1000C to 92/1000C) (Figure 5d). The reason for this trend is the change in the balance between chain growth and chain-walking, which shifts towards chain growth as the monomer concentration increases.

### 3.4. Effect of Reaction Temperature on 1-Hexene Polymerization

**Figure 6:**
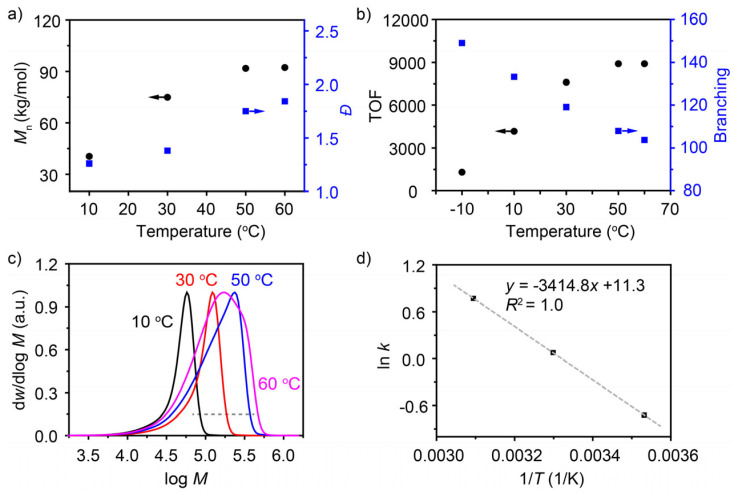
Polymerization of 1-hexene under different reaction temperatures. (**a**) Dependence of the number-average molecular weight and dispersity of the polymers on the polymerization temperature. (**b**) The change in TOF (calculated after reaction termination) and polymer branching with polymerization temperature. (**c**) GPC curves of poly(1-hexene)s. (**d**) Arrhenius plot for 1-hexene polymerization (coordination–insertion rate constants at 10, 30, and 50 °C were used for linear fitting). Polymerization conditions: polymerization temperatures are −10, 10, 30, 50, 60 °C; polymerization time: 60, 10, 10, 10, 10 min, [1-hexene] = 2 M, Ni catalyst 10 μmol, MAO (Al/Ni = 230), toluene, total volume = 40 mL.

Late transition metal catalysts are typically highly sensitive to polymerization temperature. Elevated temperatures can lead to catalyst deactivation, thereby reducing polymerization activity [30,31]. In the polymerization of 1-hexane utilizing a Ni catalyst, the number-average molecular weight of the resultant polymers displayed an initial increase with rising temperature, followed by stabilization at elevated temperatures (Figure 6a). A maximum value of approximately 92,000 g mol^−1^ was observed at 50 °C and 60 °C. The dispersities continuously increased with the reaction temperature. The GPC curves showed that the values of the higher-molecular-weight part (on the right side of the GPC peak) grew as the temperature rose, suggesting an increase in the chain growth rate. Additionally, the integral area corresponding to the lower-molecular-weight part (on the left side of the GPC peak) increased with temperature. This increase may be attributed to the intensification of the chain stagnation process at elevated temperatures [32,33]. The TOF change with temperature followed a trend analogous to that of the number-average molecular weight (Figure 6b). Random values taken from the right side of the peak in the GPC spectra were used to calculate the activation energy of the coordination–insertion process. The d*w*/dlog *M* value at 0.15 was utilized, and three sets of temperatures (10 °C, 30 °C and 50 °C) were applied to determine the activation energy (Figure 6c). By fitting the Arrhenius equation ($\ln k\;=\;-\frac{E_{a}}{R}\;\cdot \;\frac{1}{T}+\ln A$), the following linear relationship was obtained: $\ln k\;=\;-3414.8\;\cdot \;\frac{1}{T}+11.3$ ($R^{2}=1.0$) (Figure 6d). Accordingly, the average activation energy for the monomer coordination–insertion step was determined to be 28.40 kJ·mol^−1^.

In addition, the branching degree of the polymers decreased with an increase in temperature (from 149 to 103.8). This is due to the fact that while raising the temperature enhances both chain growth and chain-walking processes, the balance between the two shifts in favour of the chain-walking process [24].

### 3.5. Effect of Monomer Type on α-Olefin Polymerization

**Figure 7:**
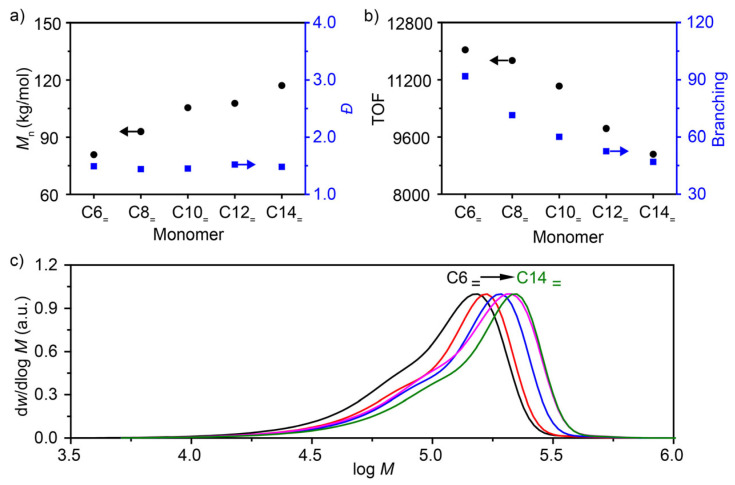
The polymerization of 1-hexene (C6_=_), 1-octene (C8_=_), 1-decene (C10_=_), 1-dodecene (C12_=_), and 1-tetradecene (C14_=_). (**a**) Dependence of the number-average molecular weight and dispersity of polymers on the monomer type; (**b**) the change in TOF (calculated after reaction termination) and polymer branching with monomer type; (**c**) GPC curves of poly(α-olefins)s. Polymerization conditions: 30 °C, 10 min, Ni catalyst 10 μmol, [monomer] = 3 M, Al_2_Et_3_Cl_3_ (Al/Ni = 200), toluene, total volume = 40 mL.

The polymerization process and the resulting product structures (branched structures containing C*_ω_*_-2_) are influenced by the chain lengths of the α-olefin monomers employed. This study utilized the α-diimine Ni catalyst/Al_2_Et_3_Cl_3_ system to investigate the polymerization of 1-hexene, 1-octene, 1-decene, 1-dodecene, and 1-tetradecene. The results indicated that all polymerization systems exhibited good catalytic activity. As the chain length of the monomer increased, the number-average molecular weight of the prepared polymers gradually increased from 80,800 to 117,100 g mol^−1^ (Figure 7a,c). In contrast, the molecular weight dispersity remained unchanged (*Ɖ*~1.5), and the TOF for the monomers decreased. Therefore, the coordination–insertion rate diminished as the monomer chain length increased. The branching degree of the products decreased from 92/1000C for poly(1-hexene) to 46/1000C for poly(1-tetradecene). The proportion of these values relative to the theoretical maxima increased from 55% to 65% (Figure 7b).

## 4. Conclusions

The polymerization of α-olefins using the classic α-diimine Ni catalyst was investigated in detail. By analyzing the polymerization kinetics, the quasi-living characteristics of the polymerization process were revealed. The reaction demonstrated living features during the initial 10 min of polymerization and gradually deviated from typical living polymerization behaviour as the reaction time progressed. A kinetic equation for the polymerization was established, showing a coordination–insertion rate constant of 1.08 L·mol^−1^·s^−1^ at 30 °C. Furthermore, the factors influencing the polymerization process and the chain structure of polymers were examined. Results from ^1^H NMR analyses, along with assessments of co-catalyst (MAO) dosage variations, showed no evidence for the *β*-H elimination or chain transfer occurring during the polymerization. The chain stagnation process is speculated to be the primary reason for the deviation from the typical living polymerization. As the monomer concentration increased, notable enhancements were observed in the molecular weight, molecular weight distribution, and branching degree of the synthesized polymer. The growth process of individual polymer chains conformed to the typical living polymerization behaviour. An increase in polymerization temperature led to an initial rise in the molecular weight of polymers, which then stabilized, while the molecular weight distribution continued to broaden and the branching degree decreased. The activation energy for monomer coordination–insertion was calculated to be 28.40 kJ·mol^−1^. Moreover, with the increase in the monomer chain length, the molecular weight of the polymers increased, while the molecular weight distribution remained unchanged, and the branching degree decreased. The insights gained from quasi-living polymerization characteristics and controllable chain branching behaviour provide a foundation for the strategic design of poly(α-olefin)s with fascinating structures and functions.

## Figures and Tables

**Table 1:** A comparison of poly(1-octene)s synthesized using metallocene Zr and α-diimine Ni catalysts.

Catalyst Structure	Chain Structure	*M*_n_ (g/mol)	*M*_w_ */M*_n_	Branches */*1000C	*T*_m_ (°C)	*T*_g_ (°C)	Usage	Ref.
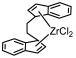	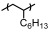	2736	2.15	125	a	a	Lubricant Base Oil	[10]
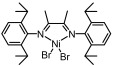	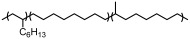	230,000	1.84	71	39	−55	Thermoplastic elastomers	[11]

a: Not detected.

## Data Availability

The original contributions presented in this study are included in the article. Further inquiries can be directed to the corresponding author.

## References

[1] Dau H., Jones G.R., Tsogtgerel E., Nguyen D., Keyes A., Liu Y.-S., Rauf H., Ordonez E., Puchelle V., Alhan H.B. (2022). Linear block copolymer synthesis. Chem. Rev..

[2] Wang Y., Lai J., Gou Q., Gao R., Zheng G., Zhang R., Song Z., Yue Q., Guo Z. (2025). Development of well-defined olefin block (co)polymers achieved by late transition metal catalysts: Catalyst, synthesis and characterization. Coord. Chem. Rev..

[3] Zhang Y.J., Li H.Y., Qu M.J., Feng N., Yang W., Zhang C. (2016). Well-defined polyolefin graft copolymers: Syntheses, structures, and properties. Prog. Chem..

[4] Chung T.C. (2002). Synthesis of functional polyolefin copolymers with graft and block structures. Prog. Polym. Sci..

[5] Tan C., Si G., Zou C., Chen C. (2025). Functional polyolefins and composites. Angew. Chem. Int. Ed..

[6] Zhu L., Yu H., Wang L., Xing Y., Ul Amin B. (2021). Advances in the synthesis of polyolefin elastomers with “chain-walking” catalysts and electron spin resonance research of related catalytic systems. Curr. Org. Chem..

[7] Zhang Y.X., Jian Z.B. (2020). Comprehensive picture of functionalized vinyl monomers in chain-walking polymerization. Macromolecules.

[8] Guo L.H., Dai S.Y., Sui X.L., Chen C.L. (2016). Palladium and nickel catalyzed chain walking olefin polymerization and copolymerization. ACS Catal..

[9] Zhang Y., Zhang Y., Hu X., Wang C., Jian Z. (2022). Advances on controlled chain walking and suppression of chain transfer in catalytic olefin polymerization. ACS Catal..

[10] Xu J., Hu Q.D., Li J.S. (2024). Performance of aromatic amine-modified metallocene polyalphaolefin lubricant base oil. Lubricants.

[11] Leone G., Mauri M., Pierro I., Ricci G., Canetti M., Bertini F. (2016). Polyolefin thermoplastic elastomers from 1-octene chain-walking polymerization. Polymer.

[12] Xia J., Kou S., Mu H., Jian Z. (2022). Slow-chain-walking polymerization of ethylene and highly chain-straightening polymerization of 1-hexene to access semicrystalline polyolefins. Eur. Polym. J..

[13] Vaidya T., Klimovica K., LaPointe A.M., Keresztes I., Lobkovsky E.B., Daugulis O., Coates G.W. (2014). Secondary alkene insertion and precision chain-walking: A new route to semicrystalline “polyethylene” from α-olefins by combining two rare catalytic events. J. Am. Chem. Soc..

[14] Pierro I., Zanchin G., Parisini E., Marti-Rujas J., Canetti M., Ricci G., Bertini F., Leone G. (2018). Chain-walking polymerization of α-olefins by α-diimine Ni(II) Complexes: Effect of reducing the steric hindrance of ortho- and para-aryl substituents on the catalytic behavior, monomer enchainment, and polymer properties. Macromolecules.

[15] McCord E.F., McLain S.J., Nelson L.T.J., Ittel S.D., Tempel D., Killian C.M., Johnson L.K., Brookhart M. (2007). ^13^C NMR analysis of α-olefin enchainment in poly(α-olefins) produced with nickel and palladium α-diimine catalysts. Macromolecules.

[16] Ruiz de Ballesteros O., Auriemma F., Guerra G., Corradini P. (1996). Molecular organization in the pseudo-hexagonal crystalline phase of ethylene−propylene copolymers. Macromolecules.

[17] Pierro I., Leone G., Zanchin G., Canetti M., Ricci G., Bertini F. (2017). Polyolefin thermoplastic elastomers from 1-octene copolymerization with 1-decene and cyclopentene. Eur. Polym. J..

[18] Leone G., Mauri M., Bertini F., Canetti M., Piovani D., Ricci G. (2015). Ni(Ii) α-diimine-catalyzed α-olefins polymerization: Thermoplastic elastomers of block copolymers. Macromolecules.

[19] Wang C.Q., Kang X.H., Dai S.Y., Cui F.C., Li Y.Q., Mu H.L., Mecking S., Jian Z.B. (2021). Efficient suppression of chain transfer and branching via *C*_s_-type shielding in a neutral nickel(II) catalyst. Angew. Chem. Int. Ed..

[20] Rhinehart J.L., Brown L.A., Long B.K. (2013). A robust Ni(II) α-diimine catalyst for high temperature ethylene polymerization. J. Am. Chem. Soc..

[21] Liu J., Chen D.R., Wu H., Xiao Z.F., Gao H.Y., Zhu F.M., Wu Q. (2014). Polymerization of α-olefins using a camphyl α-diimine nickel catalyst at elevated temperature. Macromolecules.

[22] Killian C.M., Tempel D.J., Johnson L.K., Brookhart M. (1996). Living polymerization of α-olefins using Ni^II^-α-diimine catalysts. synthesis of new block polymers based on α-olefins. J. Am. Chem. Soc..

[23] Peruch F., Cramail H., Deffieux A. (1999). Kinetic and UV−Visible spectroscopic studies of hex-1-ene polymerization initiated by an α-diimine-[N, N] nickel dibromide/MAO catalytic system. Macromolecules.

[24] Liu F.S., Gao H.Y., Hu Z.L., Hu H.B., Zhu F.M., Wu Q. (2012). Poly(1-hexene) with long methylene sequences and controlled branches obtained by a thermostable α-diimine nickel catalyst with bulky camphyl backbone. J. Polym. Sci. Part A Polym. Chem..

[25] Johnson L.K., Killian C.M., Brookhart M. (1995). New Pd(II)-based and Ni(II)-based catalysts for polymerization of ethylene and α-olefins. J. Am. Chem. Soc..

[26] Ittel S.D., Johnson L.K., Brookhart M. (2000). Late-metal catalysts for ethylene homo- and copolymerization. Chem. Rev..

[27] Gao H., Hu H., Zhu F., Wu Q. (2012). A thermally robust amine-imine nickel catalyst precursor for living polymerization of ethylene above room temperature. Chem. Commun..

[28] Nomura K., Izawa I., Yi J., Nakatani N., Aoki H., Harakawa H., Ina T., Mitsudome T., Tomotsu N., Yamazoe S. (2019). Solution XAS analysis for exploring active species in syndiospecific styrene polymerization and 1-hexene polymerization using half-titanocene-MAO catalysts: Significant changes in the oxidation state in the presence of styrene. Organometallics.

[29] Nomura K., Nagai G., Izawa I., Mitsudome T., Tamm M., Yamazoe S. (2019). XAS analysis of reactions of (arylimido)vanadium(V) dichloride complexes containing anionic NHC that contains a weakly coordinating B(C_6_F_5_)_3_ moiety (WCA-NHC) or phenoxide ligands with Al alkyls: A potential ethylene polymerization catalyst with WCA-NHC ligands. Acs Omega.

[30] Camacho D.H., Salo E.V., Ziller J.W., Guan Z.B. (2004). Cyclophane-based highly active late-transition-metal catalysts for ethylene polymerization. Angew. Chem. Int. Ed..

[31] Gates D.P., Svejda S.K., Onate E., Killian C.M., Johnson L.K., White P.S., Brookhart M. (2000). Synthesis of branched polyethylene using (α-diimine)nickel(II) catalysts: Influence of temperature, ethylene pressure, and ligand structure on polymer properties. Macromolecules.

[32] Wang Y., Gao R., Gou Q.Q., Lai J.J., Zhang R.D., Li X.Y., Guo Z.F. (2022). Developments in late transition metal catalysts with high thermal stability for ethylene polymerization: A crucial aspect from laboratory to industrialization. Eur. Polym. J..

[33] Zhu L., Zang D.D., Wang Y., Guo Y.T., Jiang B.Y., He F., Fu Z.S., Fan Z.Q., Hickner M.A., Liu Z.K. (2017). Insight into the mechanism of thermal stability of α-diimine nickel complex in catalyzing ethylene polymerization. Organometallics.

